# Capecitabine Can Induce T Cell Apoptosis: A Potential Immunosuppressive Agent With Anti-Cancer Effect

**DOI:** 10.3389/fimmu.2021.737849

**Published:** 2021-09-07

**Authors:** Sai Zhang, Zhenglu Wang, Shunli Fan, Tao Liu, Sei Yoshida, Shuang Yang, Lei Liu, Wen Hou, Lei Cao, Jianxi Wang, Zhuolun Song, Shanni Li, Sirui Zhang, Hao Wang, Jianghong Li, Hong Zheng, Zhongyang Shen

**Affiliations:** ^1^School of Medicine, Nankai University, Tianjin, China; ^2^Organ Transplant Department, Tianjin First Central Hospital, School of Medicine, Nankai University, Tianjin, China; ^3^Key Laboratory of Transplant Medicine, Chinese Academy of Medical Sciences, Tianjin, China; ^4^First Central Clinical Institute, Tianjin Medical University, Tianjin, China; ^5^National Health Commission’s Key Laboratory for Critical Care Medicine, Tianjin First Central Hospital, School of Medicine, Nankai University, Tianjin, China; ^6^Research Institute of Transplant Medicine, Nankai University, Tianjin, China; ^7^Tianjin Key Laboratory for Organ Transplantation, Tianjin First Central Hospital, School of Medicine, Nankai University, Tianjin, China

**Keywords:** capecitabine, T cell, thymidylate phosphorylase, apoptosis, immunosuppression

## Abstract

Capecitabine (CAP) is now widely used in the comprehensive treatment of digestive system tumors. Some clinical observations have shown that CAP may have immunosuppressive effects, but there is still a lack of clear experimental verification. In this study, different doses of CAP were administered to normal mice by gavage. Our results confirmed that CAP did not cause myelosuppression in bone marrow tissue; CAP selectively reduced the proportion of T cells and the concentration of related pro-inflammatory cytokines, while it increased the concentration of anti-inflammatory cytokines. Thymidylate phosphorylase (TP) is the key enzyme for the transformation of CAP *in vivo*; this study confirmed that T cells express TP, but the bone marrow tissue lacks TP expression, which explains the selectivity in pharmacodynamic effects of CAP. In addition, it was confirmed that CAP can induce T cell apoptosis *in vivo* and *in vitro. In vitro* experiments showed that CAP-induced T cell apoptosis was related to TP expression, endoplasmic reticulum stress (ERS) induction, reactive oxygen species (ROS) production, and mitochondria-mediated apoptosis activation. Therefore, this study confirmed that the differential expression of TP in cells and tissues explains why CAP avoids the toxic effects of myelosuppression while inducing T cell apoptosis to exert the immunosuppressive effect. Therefore, CAP may become an immunosuppressive agent with a simultaneous anti-cancer effect, which is worthy of further studies.

## Introductions

Capecitabine (CAP) is a classic antimetabolic chemotherapeutic drug, which has become the first-line chemotherapeutic drug for malignant tumors, such as colorectal cancer (1, 2). Recent studies have confirmed that CAP also has a good therapeutic effect on hepatocellular carcinoma (HCC) (3–6). In 2017, Ravaioli et al. found that CAP metronomic chemotherapy achieved a 1-year survival rate similar to sorafenib, and that there was no acute rejection during treatment in patients with HCC recurrence after liver transplantation (3). Their findings suggested that CAP may be a potential drug with both immunosuppressive and anti-cancer effects, which has important clinical application value in liver transplantation patients with HCC. As the oral prodrug of 5-fluorouracil (5-FU), CAP is completely absorbed through the intestinal tract; it depends on carboxylesterase (CES) and cytidine deaminase (CDA) to be converted into 5′-deoxy-5-fluorocytidin (5′-DFCR) and 5′-deoxy-5-fluorouridine (5′-DFUR), which are finally converted to 5-FU by thymidylate phosphorylase (TP). CES and CDA are highly expressed in the liver, which may endow CAP-related intermediate metabolites with a higher concentration in the liver (7). Moreover, the expression of TP in the cancer tissue is significantly higher than that in the surrounding normal tissues; thus, a higher concentration of 5-FU accumulates in cancer tissue and exerts an anti-cancer effect (8). Therefore, the distribution of TP determines the distribution of roles of CAP. Since TP is also highly expressed in lymphocytes (9), another characteristic of CAP is the ability to target cancer cells and lymphocytes simultaneously. These two pharmacological characteristics provide potential advantages and theoretical basis for us to explore the application of CAP as a drug with both immunosuppressive and anti-cancer effects in liver transplantation patients with HCC.

Previous studies have confirmed that 5-FU—the active component of CAP *in vivo*—nonselectively inhibits immune cells, such as T cells, B cells, NK cells, and monocytes in peripheral blood of mice (10); at the same time, 5-FU also has a severe myelosuppression effect (11–13). In contrast, due to the difference in the distribution of TP in tissues and cells, CAP may circumvent some of the side effects of 5-FU; thus, it is possible that its impact on the immune system may be different from that of 5-FU. The current research on the effect of CAP on immunity is limited to related research on CAP combined with other chemotherapeutic drugs in the field of tumor immunity (14–17). However, there is still a lack of experimental observations on the effect of CAP monotherapy on the lymphocyte subsets, cytokines, and bone marrow of normal mice. Therefore, here, we constructed different doses of CAP mice feeding models to observe the effects of CAP on the immune system of mice, with the idea to provide the basis for understanding and supporting the use of CAP as an immunosuppressive agent with anti-cancer effects to optimize the medication regimen in liver transplant patients with HCC in the future.

## Materials and Methods

### Animals

All the animals were obtained from China National Institutes for Food and Drug Control. Male Balb/c mice, aged 6–8 weeks (20–22 g) were housed in a room with a 12 h light/dark cycle with access to standard laboratory food and filtered clean water *ad libitum*. After one-week acclimatization to the laboratory conditions, the mice were assigned into three equal groups as follows: (i) CON group (n=15): intragastric 0.9% normal saline; (ii) MET group (n=15): intragastric metronomic dose of capecitabine (Solarbio, Beijing, China) (100 mg/kg·day) (16); (iii) MTD group (n=15): intragastric maximum tolerated dose of capecitabine (Solarbio, Beijing, China) (400 mg/kg·day) (16). On the 7th, 14th, and 21st day after gavage, five mice were sacrificed at one time. The animals received humane care and were maintained in accordance with the guidelines established by the Committee on Laboratory Resources, National Institutes of Health. All the experiments were approved by the Ethics Committee of Nankai University.

### Cell Lines

The T lymphoma cell lines EL4 and MOLT4 were purchased from the National Collection of Authenticated Cell Cultures (Shanghai, China). EL4 and MOLT4 cells were cultured in RPMI medium 1640 and DMEM medium (Gibco, Grand Island, CA, USA) supplemented with 10% fetal bovine serum (FBS) (Biowest, Loire Valley, France), 100 IU/mL penicillin, and 100 μg/mL streptomycin (Solarbio, Beijing, China) and maintained in a humidified incubator (37°C, 5% CO_2_).

### Flow Cytometry Analysis

Mononuclear cells of spleen and peripheral blood were isolated from the sacrificed mice using mouse organ mononuclear cells separation solution (Solarbio, Beijing, China) by gradient centrifugation. The purified mononuclear cells were incubated with anti-CD3, anti-CD4, anti-CD8, anti-CD19, or anti-CD49b antibodies (BioLegend, San Diego, CA, USA). At least 5000 mononuclear cells per sample were acquired, and the absolute count of lymphocyte subset per 5000 cells was calculated based on Accuri C6 Plus flow cytometry (BD Biosciences, Palo Alto, CA, USA) with positive markers: CD3^+^ T cells, CD3^+^CD4^+^ T cells, CD3^+^CD8^+^ T cells, CD19^+^ B cells, and CD49b^+^ NK cells.

### Luminex

The concentrations of serum cytokines (CCL2, CCL3, CCL4, CCL5, CCL20, CXCL1, CXCL2, G-CSF, GM-CSF, M-CSF, IFN-γ, IL-1α, IL-1β, IL-2, IL-4, IL-5, IL-6, IL-10, IL-12, IL-13, IL-17, VEGF, and TNF-α) were detected with LXSAHM-23 (R&D Systems Inc., Minnesota, USA) following the manufacturer’s instructions. Briefly, serum samples were incubated with antibodies conjugated to microspheres, biotinylated antibodies and streptavidin–phycoerythrin fluorescent conjugate (SA-PE) in sequence. The Luminex ^®^ 200TM instrument was used to detect the intensity of the signal for each microsphere added to the protein samples.

### Cell Viability

The cell viability was evaluated by CCK-8 assay (Boster, Hubei, China) and calculated as the percentage of (OD test − OD blank)/(OD control − OD blank). Then, the half-maximal inhibitory concentration (IC50) was calculated by using GraphPad 8.0 software (GraphPad Software Inc., CA, USA) based on the data of CCK-8 assay.

### Hematoxylin and Eosin Staining

The femora were decalcified by EDTA decalcifying solution (Solarbio, Beijing, China) at first. Then, the femora were fixed with 10% neutral formalin solution, dehydrated, paraffin embedded, sliced into 4-µm sections, and stained with H&E in sequence. The morphological changes in the bone marrow were evaluated using a Ni-U microscope (Nikon, Japan) at 100× magnification.

### Immunohistochemistry Assay

Briefly, the samples were dehydrated, embedded in paraffin, sectioned, and subjected to TP and CD34 (Abcam, Cambridge, UK) antibodies. The signal was obtained and photographed under a microscope at 200× magnification.

### Isolation of CD3^+^T Cells and Activation

Mononuclear cells from the spleen of mice were collected, and CD3^+^ T cells were isolated using anti-CD3 microbeads (Miltenyi Biotec, Germany). CD3^+^ T cells were cultivated in 24-well plates at 1×10^6^ cells/mL per well in a culture medium with anti-CD3 antibody (2 µg/mL) and anti-CD28 antibody (1 µg/mL). After 16–18 h, the cells were collected for further use.

### Apoptosis Assays

An apoptosis kit (Solarbio, Beijing, China) was employed to detect apoptosis of T cells in line with the manufacturer’s instructions. Apoptotic cells were included the sum of early (Annexin V^+^PI^−^) and late apoptotic (Annexin V^+^PI^+^) cells.

### Measurement of Intracellular ROS Levels

Accumulation of intracellular ROS was determined by flow cytometry using the Reactive Oxygen Species Assay Kit (Solarbio, Beijing, China). In brief, the cells were collected and resuspended in a serum-free medium containing 10μM DCFH-DA. DCF fluorescence intensity was then detected by flow cytometry.

### Measurement of Mitochondrial Membrane Potential

The mitochondrial membrane potential in T cells was detected using mitochondrial membrane potential assay kit with JC-1 (Solarbio, Beijing, China) in accordance with the manufacturer’s instructions. Cells were incubated with JC-1 working solution for 20 min at 37°C in the dark; then, they were washed twice with JC1 buffer solution before the flow cytometry analysis.

### Western Blotting

Isolation of mitochondria and cytosolic fractions was conducted with the Mitochondria Fractionation Kit (Solarbio, Beijing, China) following the manufacturer’s instructions. Bone marrow cells were collected from the femora. Total proteins extracted were analyzed by western blotting as previously described (18). The expression levels of caspase-3, ATF4, GRP78, CHOP, TP (Santa Cruz, CA, USA), PERK, p-PERK (Immunoway, Texas, USA), BAX, Bcl2, and Cytochrome C (Abcam, Cambridge, UK) were detected. GAPDH, β-actin, and Cox IV (SAB, Maryland, USA) antibodies were used as the internal controls. The membranes were scanned with an imaging system (Bio-Rad, Hercules, CA, USA), and the bands were analyzed using ImageJ 7.0 software (National Institutes of Health, USA).

### Statistical Analysis

SPSS 13.0 (SPSS GmbH, Munich, Germany) and GraphPad 8.0 were used for statistical analysis. Data were expressed as mean ± standard deviation (SD). Student’s *t*-test was used to determine differences between two groups, and one-way analysis of variance (ANOVA) was used to determine the differences among three or more groups. The *p* value below 0.05 was considered to be statistically significant.

## Results

### The Lack of TP Expression in Bone Marrow Circumvents the Side Effects of Myelosuppression by CAP

**Figure 1:**
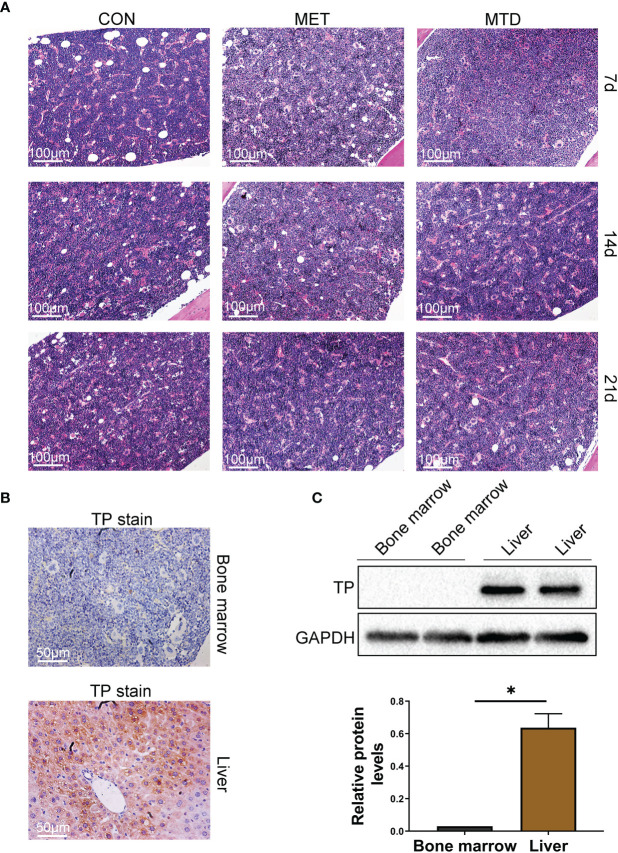
Pathological changes and TP expression in bone marrow of mice. **(A)** Bone marrow tissue was stained with H&E (100×). CON: control groups, in which mice received 0.9% normal saline. MET: CAP-treated groups in which mice received metronomic chemotherapy dose of CAP (100 mg/kg/d). MTD: CAP-treated groups in which mice received maximum tolerated dose of CAP (400 mg/kg/d). **(B)** TP in bone marrow and liver was stained with IHC (200×). TP in liver served as a positive control. **(C)** Bone marrow cells were collected from the femora, protein levels of TP were evaluated using the western blot assay, and TP in the liver served as a positive control. Data are shown as mean ± SD. *P < 0.05.

In order to solve the clinical problem of HCC recurrence after liver transplantation, we tried to find a drug with both immunosuppressive and anti-cancer effects. Some clinical observations have shown that CAP, a classic chemotherapy drug, may have immunosuppressive effects, but there is still a lack of clear experimental verification. Therefore, we constructed different doses of CAP (metronomic chemotherapy or maximum tolerated dose) mice feeding models to observe the effects of CAP on the immune system of mice. The common side effect of 5-FU (the active ingredient transformed by CAP *in vivo*) is myelosuppression (11). As shown in **Supplementary Figure S1**, 5-FU was used as a positive control, and it did cause myelosuppression in the mice. Therefore, we must first evaluate the myelosuppression effect of CAP to determine the feasibility of its long-term use. As shown in **Figure 1A**, on days 7, 14, and 21, the bone marrow of mice in the MET (metronomic chemotherapy dose) and MTD (maximum tolerated dose) groups had no pathological manifestations of myelosuppression. Compared with the CON group, there was no significant difference in the cellularity (%) of bone marrow and the expression of CD34 (the classical progenitor marker) in the MET and MTD groups on days 7, 14, and 21 (**Supplementary Figures S1****,** **S2**). These results showed that CAP did not cause significant myelosuppression. Next, we continued to seek the reasons why CAP did not cause myelosuppression. Considering that the conversion of CAP to 5-FU *in vivo* depends on TP, we speculated that the reason why CAP did not cause myelosuppression may be related to the lack of expression of TP in bone marrow tissue. Next, considering that a previous study has confirmed that TP is highly expressed in the liver (9), liver tissue was selected as the positive control, and its TP immunohistochemical (IHC) staining was performed simultaneously with that of bone marrow. As shown in **Figure 1B**, TP was expressed in the liver, but TP expression was rarely detected in bone marrow tissue (only very few cells in the bone marrow expressed TP). Subsequently, we collected bone marrow cells to detect the expression of TP by western blot. As shown in **Figure 1C**, there was no TP expression in bone marrow cells. The above results confirmed that the lack of TP expression in bone marrow tissue was responsible for circumventing the side effects of myelosuppression by CAP.

### The Influence of CAP on Lymphocyte Subsets in Mice

**Figure 2:**
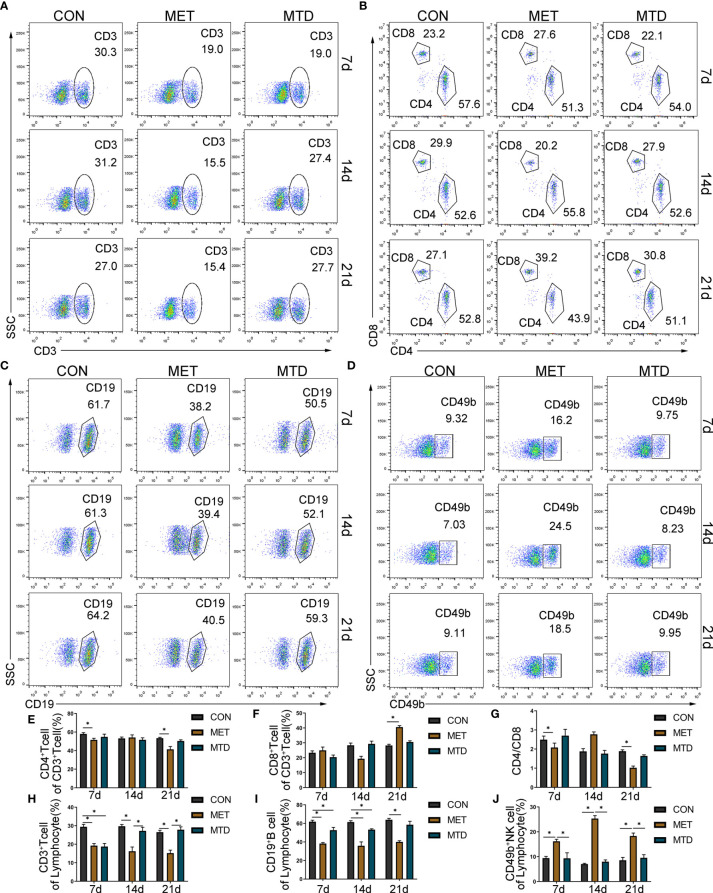
Proportion of lymphocyte subsets in the spleen of mice. The percentage of CD3^+^ T cells in lymphocyte **(A, H)**; the percentage of CD4^+^ T cells **(B, E)** and CD8^+^ T cells **(B, F)** in CD3^+^ T cells; the ratio of CD4/CD8 **(B, G)**; the percentage of CD19^+^ B cells **(C, I)** and CD49b^+^ NK cells **(D, J)** among lymphocytes, as detected by flow cytometry. CON: control groups, in which mice received 0.9% normal saline. MET: CAP-treated groups in which mice received metronomic chemotherapy dose of CAP (100 mg/kg/d). MTD: CAP-treated groups in which mice received maximum tolerated dose of CAP (400 mg/kg/d). Data are shown as mean ± SD. *P < 0.05.

Next, we used flow cytometry to detect the proportion and number of lymphocyte subsets in the spleen and peripheral blood of the mice. As shown in **Figure 2** and **Supplementary Figures S3****–****S5**, in terms of T cells and their subgroups, on days 7, 14, and 21, the proportion and number of CD3^+^ T cells in the spleen and peripheral blood were significantly lower in the MET group than in the CON group; compared with the CON group, the proportion and number of CD3^+^ T cells in the spleen and peripheral blood were significantly lower in the MTD group on day 7; compared with the MTD group, the proportion and number of CD3^+^ T cells in the spleen was significantly lower in the MET group on days 14 and 21; compared with the CON group, the proportion and number of CD4^+^ T cells as well as the ratio of CD4/CD8 in the spleen and peripheral blood were significantly lower on day 7. In terms of B cells, on days 7, 14, and 21, the proportion and number of CD19^+^B cells in the spleen and peripheral blood of the MET group were significantly lower than those of the CON group; the proportion and number of B cells in the spleen and peripheral blood of the MTD group was significantly lower than that of the CON group on day 7. In terms of NK cells, the proportion and number of NK cells in the spleen and peripheral blood of the MET group were significantly higher than those in the CON group on days 7 and 14; compared with the CON group, there was no significant difference in the MTD group. The above results indicate that metronomic chemotherapy and maximum tolerated dose of CAP can decrease the proportions and numbers of T and B cells in both spleen and peripheral blood. At the same time, the metronomic chemotherapy dose of CAP increased the proportion and number of NK cells; this effect was different from the maximum tolerated dose of CAP.

### Changes in Cytokine Concentration in Peripheral Blood of Mice

**Figure 3:**
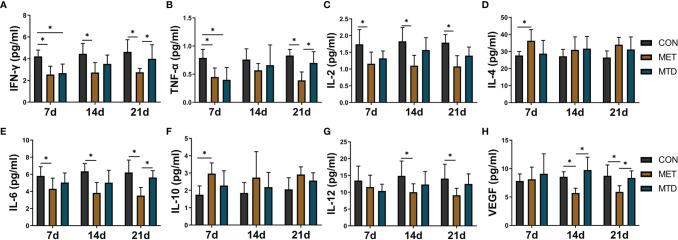
The concentrations of serum cytokines in the mice treated with different dosages of CAP. The levels of **(A)** IFN-γ, **(B)** TNF-α, **(C)** IL-2,**(D)** IL-4, **(E)** IL-6, **(F)** IL-10, **(G)** IL-12, and **(H)** VEGF were detected by Luminex assay. CON: control groups, in which mice received 0.9% normal saline. MET: CAP-treated groups, in which mice received metronomic chemotherapy dose of CAP (100 mg/kg/d). MTD: CAP-treated groups, in which mice received maximum tolerated dose of CAP (400 mg/kg/d). Data are shown as mean ± SD. ^*^P < 0.05.

To further observe the immunosuppressive effect of CAP, we focused on cytokine, which is another important aspect of the immune system. We used Luminex technology to analyze 23 cytokines in the serum of mice. Among them, the levels of IL-1α, IL-1β, IL-13, IL-17, CCL2, and CCL-20 6 cytokines were lower than the minimum detection concentration and were not detected. As shown in **Figure 3**, in the MET group, compared with the CON group, the concentrations of IL-2, IL-6, IFN-γ, and TNF-α were significantly low and the concentrations of IL-4 and IL-10 were significantly high at day 7. Compared with the CON group, the concentrations of IL-12 and VEGF were significantly lower on days 14 and 21. In the MTD group, compared with the CON group, the concentrations of IFN-γ and TNF-α were significantly lower only on day 7. Compared with the MTD group, the concentrations of IFN-γ, TNF-α, and IL-6 decreased significantly on day 21; the concentration of VEGF decreased significantly in the MET group on days 14 and 21. Other cytokines that did not show significant changes are shown in **Supplemental Figure S6**. The above results show that, at the cytokine level, metronomic chemotherapy dose of CAP also produces an immunosuppressive effect; in particular, it reduces the concentration of pro-inflammatory cytokines and increases the concentration of anti-inflammatory cytokines. At the same time, in contrast to the maximum tolerated dose of CAP, CAP reduced the concentration of VEGF at the metronomic chemotherapy dose.

### CAP Increases T Cell ROS Generation and Mitochondrial Membrane Depolarization, and Induces Apoptosis *In Vivo*

**Figure 4:**
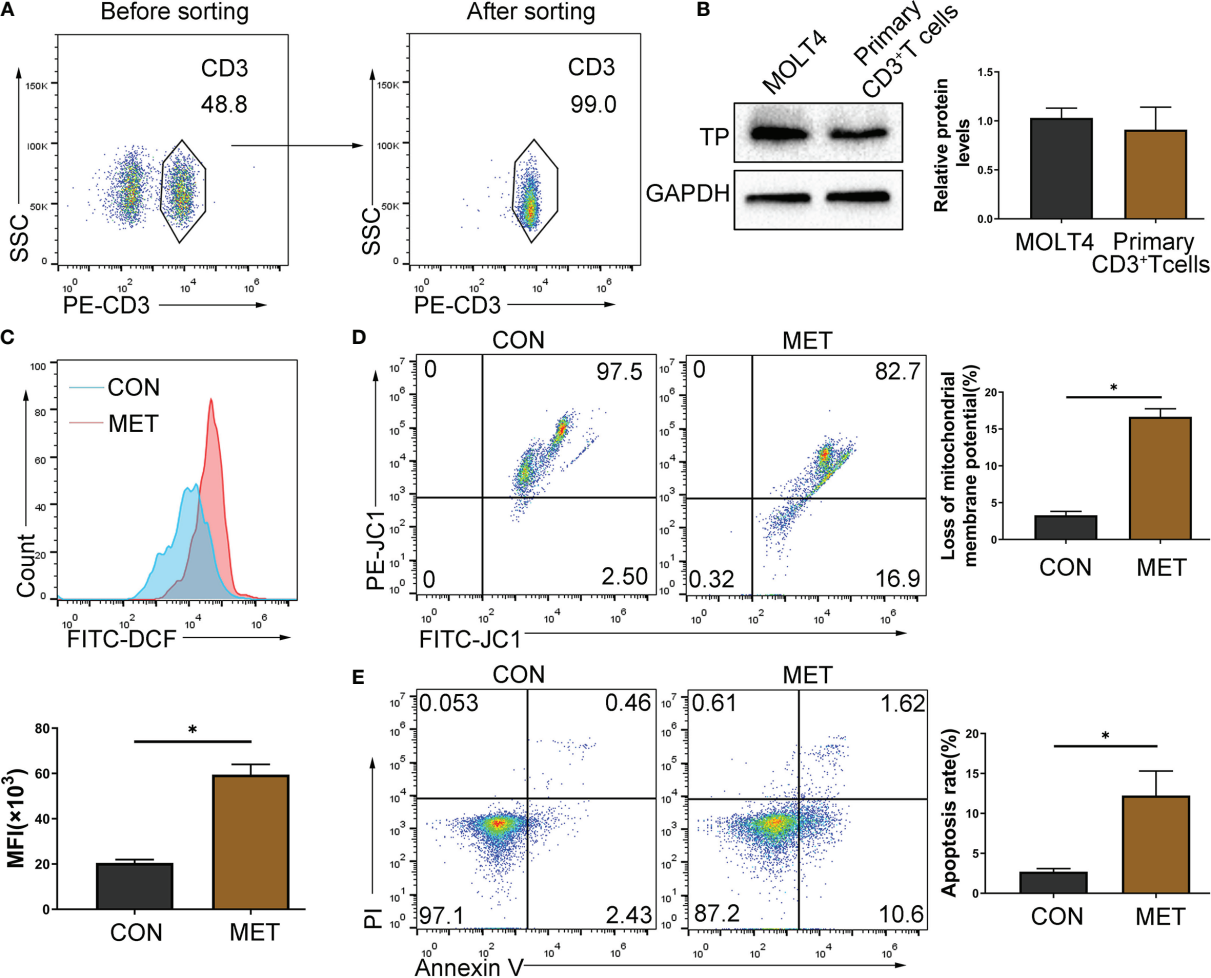
CAP can induce apoptosis of TP-expressing T cells. **(A)** CD3^+^ T cells were sorted by immunomagnetic beads from the spleen of mice and identified by staining with PE-CD3 antibody. **(B)** The protein levels of TP were evaluated using western blot assay. **(C)** Mononuclear cells extracted from the spleen of mice gavaged with metronomic chemotherapy dose of CAP on day 7 were collected and gated by CD3, and then the ROS level was detected with fluorescent probes (DCFH-DA) using flow cytometry; **(D)** The proportion of cells with reduced mitochondrial membrane potential was detected using flow cytometry; **(E)** The apoptosis of T cells was detected by Annexin V and PI staining. Data are shown as mean ± SD. ^*^P < 0.05.

T cell-mediated cellular immunity is particularly important in the acute rejection of organ transplantation, and T cells are the target immune cells often selected by clinical immunosuppressants. In this study, metronomic chemotherapy dose of CAP was shown to reduce the proportion and number of T cells. Next, we sorted mouse primary CD3^+^ T cells, and the expression of TP in T cells was confirmed by western blot (**Figures 4A, B**) (MOLT4 T cell, which has been confirmed to express TP (19), was selected as the positive control). Thus, we chose T cells to further explore the mechanism behind the immunosuppressive effect of CAP. Because 5-FU, the active ingredient of CAP *in vivo*, can induce the rise in ROS and subsequently triggers apoptosis through the mitochondrial pathway to exert antitumor effects (20), we considered that CAP may exert an immunosuppressive effect by directly inducing T cell apoptosis. Because we have confirmed that in terms of the reduction in the proportion and number of T cells, the metronomic chemotherapy dose of CAP has more advantages than the maximum tolerated dose, we chose a metronomic chemotherapy dose of CAP for further research. We used flow cytometry to detect the ROS levels and the proportion of cells with decreased mitochondrial membrane potential and apoptotic rate of CD3^+^ T cells in spleen of mice gavaged with metronomic chemotherapy dose of CAP at day 7. As shown in **Figures 4C–E**, compared with the CON group, the ROS levels, the proportion of cells with decreased mitochondrial membrane potential and the proportion of apoptotic T cells in the MET groups increased significantly. The above results show that the metronomic chemotherapy dose of CAP can increase ROS levels and reduce mitochondrial potential to induce T cell apoptosis *in vivo*.

### Differential Expression of TP in T Cells Affects CAP-Induced Apoptosis *In Vitro*

**Figure 5:**
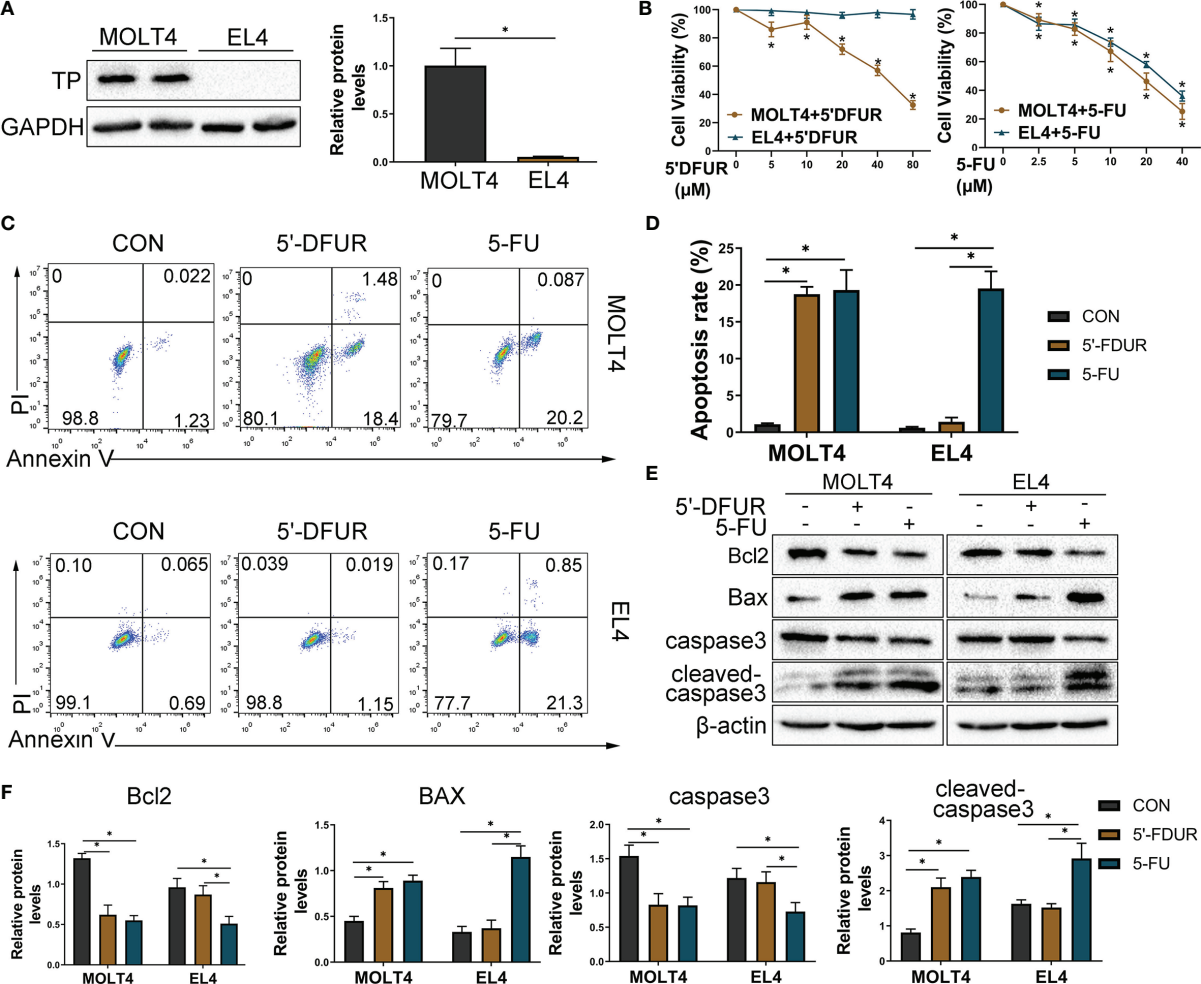
Differential expression of TP in T cells affects CAP-induced apoptosis *in vitro*. T cell lines (EL4 and MOLT4) were selected for further research *in vitro*, and we chose 5′DFUR (conversion to 5-FU depends on TP) and 5-FU, both of which are intermediate metabolites of CAP, instead of CAP for the *in vitro* experiments. **(A)** TP protein levels in EL4 and MOLT4 cells were detected by western blot analysis. **(B)** Viability of EL4 and MOLT4 cells exposed to gradient concentration of 5′-DFUR or 5-FU for 48 h was measured by CCK8 test. **(C, D)** According to IC50, EL4 and MOLT4 cells were exposed to 5′-DFUR (0 μM and 50 μM) or 5-FU (0 μM and 30 μM) for 48 h; apoptosis of T cells was detected by Annexin V/PI staining. **(E, F)** The protein levels of apoptosis-related proteins, including caspase3, Bcl2, and BAX, were evaluated using western blot assay. Data are shown as mean ± SD. ^*^P < 0.05.

**Figure 6:**
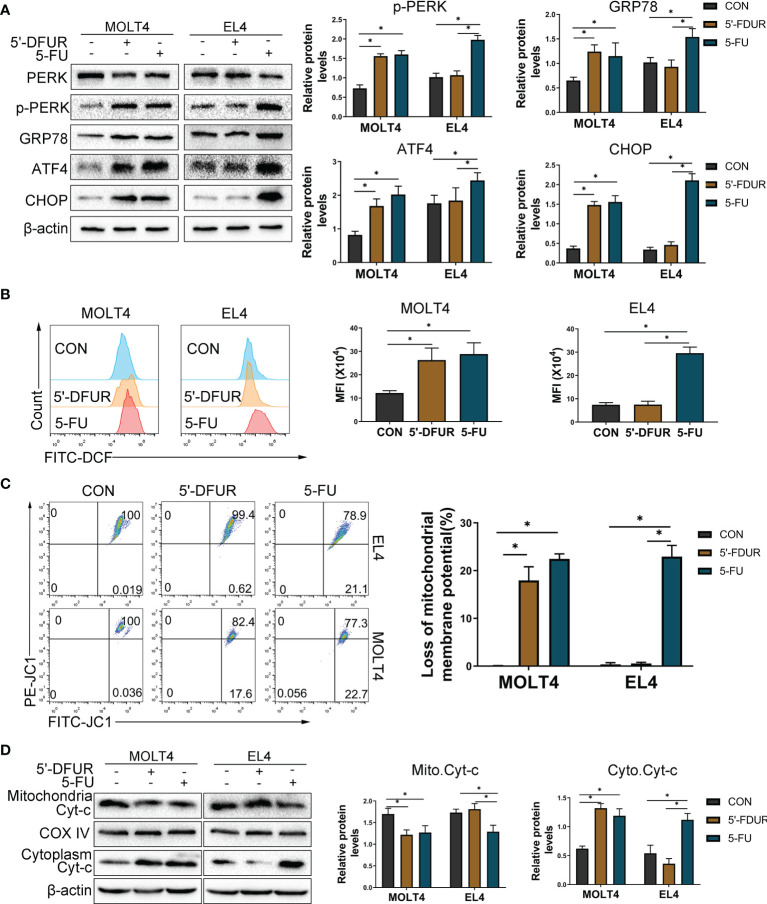
CAP increases T cell ERS, ROS generation, mitochondrial membrane depolarization, and induces mitochondria-mediated apoptosis. EL4 and MOLT4 cells were exposed to 5′-DFUR (0 μM and 50 μM) or 5-FU (0 μM and 30 μM) for 48 (h) **(A)** Endoplasmic reticulum stress-related proteins PERK, p-PERK, GRP78, ATF4, and CHOP were detected by western blot analysis. **(B)** The ROS level was detected with fluorescent probes (DCFH-DA) using flow cytometry. **(C)** The proportion of cells with reduced mitochondrial membrane potential was detected using flow cytometry. **(D)** Cytochrome C expression in the cytoplasm and mitochondria was detected by western blot analysis. Data are presented as mean ± SD. ^*^P < 0.05.

Previous studies have confirmed that 5-FU can activate the ERS and induce the increase in ROS in colon cancer cells, and subsequently induces apoptosis through the mitochondrial pathway (20). Thus, we hypothesized that ERS/ROS/mitochondria- mediated apoptosis may be the mechanism behind apoptosis of T cells induced by CAP. In this study, it has been confirmed that CAP can induce T cell apoptosis, which is related to the production of ROS levels and the decrease in mitochondrial potential *in vivo* (**Figures 4C–E**). Considering that the effect of CAP depends on the expression of TP in cells, we tried to confirm whether the expression of TP in T cells will affect the apoptosis induced by CAP. Subsequently, we selected MOLT4 T cells (expressing TP) and EL4 T cells (lack of TP expression) (**Figure 5A**) for further exploration. We chose different concentrations of two intermediate metabolites of CAP *in vivo*, 5-deoxyfluorouridine (5′-DFUR, which is converted to 5-FU by TP) and 5-FU, to culture T cells for 48 h. The cell viability of MOLT4 cells was significantly inhibited by 5-FU or 5′-DFUR (IC50: 21.8 ± 7 μM and 51.3 ± 14.4μM, respectively); in contrast, the cell viability of EL4 cells was only significantly inhibited by 5-FU (IC50: 33.4 ± 9.8 μM), while 5′-DFUR had no significant effect on their viability (**Figure 5B**). Subsequently, according to IC50, we chose 5-FU (0, 30 μM) or 5′-DFUR (0, 50 μM) to culture MOLT4 and EL4 cells for 48 h, respectively. The results of flow cytometry indicated that 5-FU induced EL4 and MOLT4 cell apoptosis, while 5′-DFUR only induced MOLT4 cell apoptosis (**Figure 5C, D**). After that, western blot assay showed that 5-FU significantly increased the expression levels of cleaved-caspase3 and pro-apoptotic protein BAX, and anti-apoptotic protein Bcl2 in EL4 and MOLT4 cells. In contrast, 5′-DFUR only caused the abovementioned changes in MOLT4 cells (**Figures 5E, F**). Next, we found that 5-FU significantly increased the expression levels of ERS-related proteins GRP78, ATF4, p-PERK, and CHOP in EL4 and MOLT4 cells; in contrast, 5′-DFUR only caused the abovementioned changes in MOLT4 cells (**Figure 6A**). At the same time, in MOLT4 cells, both 5-FU and 5′-DFUR significantly increased the ROS levels, and the proportion of cells with decreased mitochondrial membrane potential increased significantly. The expression of mitochondrial cytochrome C was significantly reduced, and the expression of cytochrome C in the cytoplasm significantly increased. However, in EL4 cells, only 5-FU, but not 5′-DFUR, caused the above-mentioned changes (**Figures 6B–D**). The above results confirmed that the apoptosis induced by CAP in T cell is also related to TP expression, ERS induction, ROS production and mitochondria-mediated apoptosis activation.

### Inhibition of ERS Attenuates CAP-Induced Primary T Cells Apoptosis

**Figure 7:**
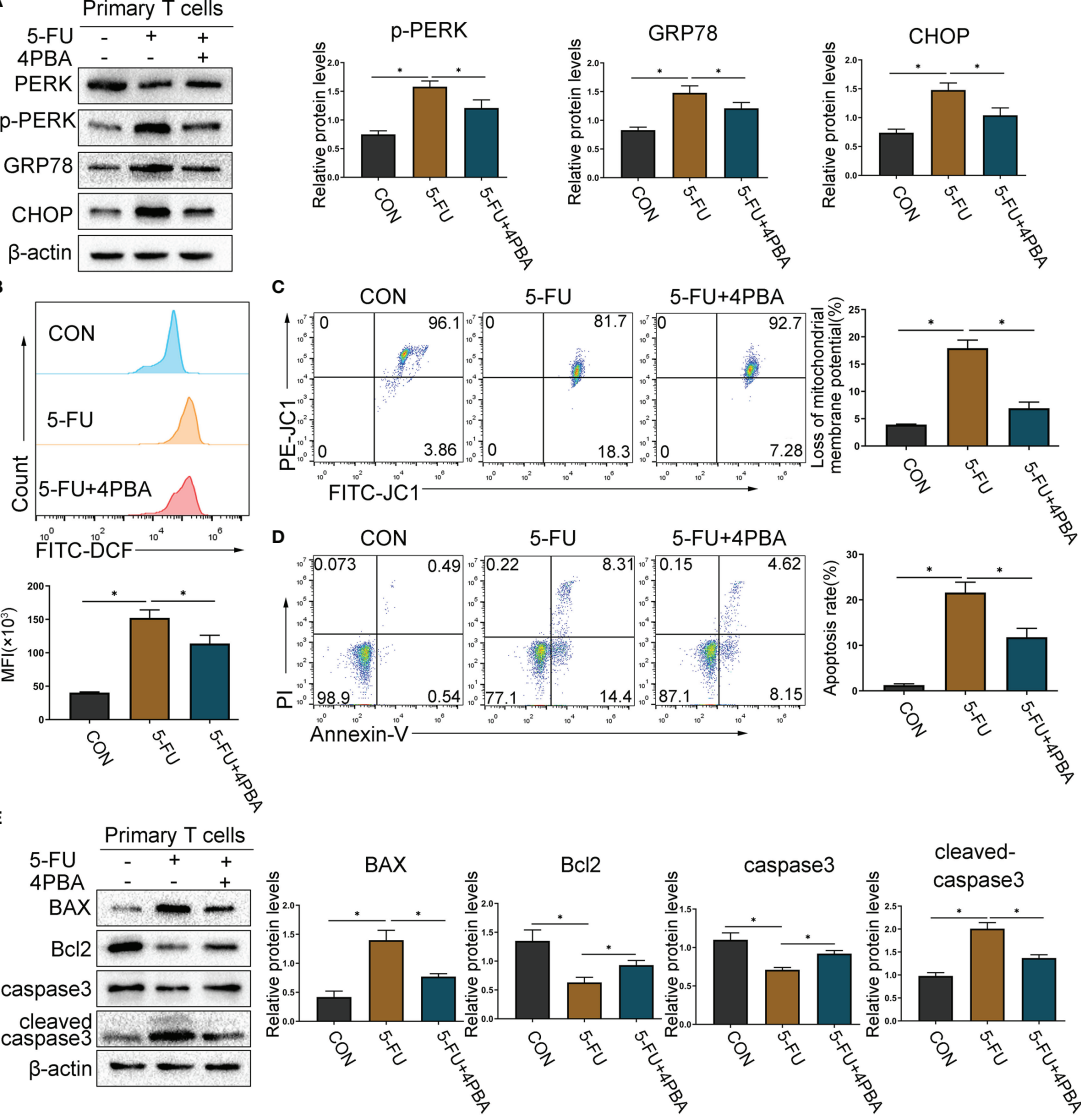
Inhibition of ERS attenuates CAP-induced primary T cells apoptosis. CD3^+^ T cells were sorted by magnetic beads isolation and *in vitro* stimulation with anti-CD3/CD28 antibodies. Primary T cells were exposed to 5-FU (0 μM and 10 μM) or 5-FU (10 μM) + 4PBA (1000 μM) for 48 h. **(A)** ERS-related proteins PERK, p-PERK, GRP78, and CHOP were detected by western blot analysis. **(B)** The ROS level was detected with fluorescent probes (DCFH-DA) using flow cytometry. **(C)** The proportion of cells with reduced mitochondrial membrane potential was detected using flow cytometry. **(D)** The apoptosis of T cells was detected by Annexin V and PI staining. **(E)** The protein levels of apoptosis-related proteins, including caspase3, Bcl2, and BAX, were evaluated using western blot assay. Data are shown as mean ± SD. ^*^P < 0.05.

Taking into account the differences between T cell lines and primary T cells, we sorted mouse spleen CD3^+^ T cells to further explore the mechanism of CAP-induced T cell apoptosis. CD3^+^ T cells were sorted by magnetic beads isolation and *in vitro* stimulation with anti-CD3/CD28 antibodies. At first, we chose different concentrations of 5-FU to culture primary T cells for 48 h. The cell viability of primary T cells was significantly inhibited by 5-FU (IC50: 11.3 ± 2.54 μM) (**Supplementary Figure S7A**). Subsequently, we chose different concentrations of 4-Phenylbutyric acid (4PBA), which is an ERS inhibitor, to culture primary T cells for 48 h. CCK8 results (**Supplementary Figure S7B**) showed that there was no significant change in primary T cells viability. Next, according to IC50 of 5-FU, we chose 5-FU (10 μM) or 5-FU (10 μM) + 4PBA (0 μM–10000 μM) to culture primary T cells for 48 h, in order to determine the appropriate concentration of 4PBA for further experiments. As shown in (**Supplementary Figure S7B**), compared with 5FU (10 μM), 5-FU (10 μM) + 4PBA (1000 μM) significantly increased the vitality of primary T cells. So, we chose 5-FU (10 μM) and 4PBA (1000 μM) for further experiments. As shown in (**Figures 7A–E**), compared with the CON group, 5-FU significantly increased primary T cells ERS, ROS generation, mitochondrial membrane depolarization, and induced apoptosis. Compared with the 5-FU group, using 4PBA to reduce ERS can significantly reduce ROS generation, mitochondrial membrane depolarization, and induction of apoptosis by 5-FU. The above results confirmed that ERS/ROS/mitochondria-mediated apoptosis may be the mechanism behind apoptosis of primary T cells induced by CAP.

## Discussion

The recurrence of HCC after liver transplantation has become a key clinical problem to be solved urgently (21, 22). Thus, finding a drug with both immunosuppressive and anti-cancer effects has important clinical application value. As a classic chemotherapeutic drug, a large number of clinical studies have confirmed the efficacy of capecitabine in the field of HCC (4, 6). Moreover, there is evidence that CAP may have an immunosuppressive effect, but this still needs to be confirmed by experiments (3). Therefore, we chose metronomic chemotherapy (a new type of chemotherapy featuring low-dose, uninterrupted, and continuous administration) dose or maximum tolerated dose of CAP to treat normal mice by oral gavage to explore the feasibility of CAP as a potential immunosuppressive agent (23).

First, considering myelosuppression, the common side effect of 5-FU (the active ingredient transformed by CAP *in vivo*) (11, 24), we must evaluate whether CAP also causes myelosuppression, so as to determine the feasibility of its long-term application. In this study, no significant myelosuppression was observed in either the metronomic chemotherapy dose or maximum tolerated dose of CAP. The lack of TP expression in bone marrow explains why CAP does not cause myelosuppression there. Next, at the level of lymphocyte subsets, CAP, especially in the metronomic chemotherapy dose, reduced the number of CD3^+^ T cells. The cellular immunity mediated by T cells is the immune basis for the acute rejection of organ transplantation (25, 26). Immunosuppressants commonly used in clinical practice mainly target T cells to prevent rejection (27, 28). In addition to the reduction of T cells, the number of B cells also declined significantly. As another important adaptive immune cell, B cells differentiate into plasma cells with the help of antigen stimulation and Th cells and produce specific immunoglobulins to participate in humoral immunity (29). Recent studies have suggested that during the development of allogeneic liver transplantation rejection, the role of B cells in presenting donor antigens is enhanced (30). Certainly, considering the importance of T and B cells in maintaining normal cellular immunity and humoral immune function (31, 32), it should be investigated whether lymphopenia caused by CAP may affect physiological immune function. Meanwhile, considering that lymphopenia and immunosuppression are closely related but different, possible roles of CAP in regulating the functions of T and B cells after activation also merit further investigation. In addition to T and B cells, the metronomic chemotherapy dose of CAP significantly increased the number of NK cells, which is similar to the results of the previous study (14). Even if the role of NK cells as classical innate immune cells in acute rejection is still unclear, considering the importance of NK cells in tumor immunotherapy (33), this may be the advantage of the anti-cancer effect of the metronomic chemotherapy dose of CAP. However, in the context of lymphopenia caused by CAP, further experimental confirmation as to whether metronomic chemotherapy dose of CAP can truly activate tumor immunity is needed. At the cytokine level, the metronomic chemotherapy dose of CAP significantly reduced the concentration of proinflammatory cytokines that activate the immune response (IL-2, TNF-α, IFN-γ, IL-6, IL-12), and increased the concentration of certain anti-inflammatory cytokines with immunosuppressive characteristics (IL4, IL-10). IL-2, TNF-α, IFN-γ, and IL-6 are mainly secreted by T cells, and participate in a broad array of immune responses (34–38). IL4 and IL10 can regulate the transformation of Th0 to Th2 and the release of proinflammatory cytokines to induce immune tolerance (39–41). During the experiment, an interesting phenomenon was also observed. It seems that the metronomic chemotherapy dose of CAP is more advantageous than the maximum tolerated dose of CAP, both at the level of lymphocyte subsets and at the level of cytokines late in the experiment. We speculate that these findings might be related to the dose of CAP. Namely, metronomic chemotherapy dose of CAP is suitable for long-term continuous use due to low dose and low adverse effects; in contrast, the maximum tolerated dose (conventional chemotherapy dose) of CAP is not suitable for long-term continuous application due to its side effects (42, 43). In this experiment, the mice may not be able to tolerate the toxicity of high-dosage long-term treatment with CAP, and the possible toxic side effects of high-dose CAP may have partially counterbalanced the immunosuppressive effects. However, further studies are required to verify this hypothesis. Still, the results from both the mouse experiment and clinical study suggest that metronomic chemotherapy dose of CAP may be more suitable for further study as a potential immunosuppressive agent.

Next, to explore the mechanism behind the immunosuppressive effect of CAP, we chose T cells for further studied because T cells play an important role in acute rejection after transplantation and are the main target cells of immunosuppressive agents commonly used in clinical (25, 28). As a classic chemotherapeutic drug, CAP is converted into 5′-DFUR; then, 5′-DFUR is converted to 5-FU through TP, and 5-FU can induce tumor cell apoptosis to exert anti-cancer effect (20, 44). Therefore, the induction of T cells apoptosis may be the underlying mechanism of CAP’s immunosuppressive effect, and the presence of TP in T cells may be the basis for CAP-induced T cells apoptosis. In this study, TP expression in T cells was validated, and CAP was shown to induce T cell apoptosis *in vivo* and *in vitro*. At the same time, *in vitro* experiments showed that 5′-DFUR induced apoptosis in TP-expressing T cells, but not in TP-lacking T cells. The reason may be that 5′-DFUR cannot be further converted into 5-FU in T cells lacking TP expression. Then, we further explored the mechanism of CAP-induced T cell apoptosis. The endoplasmic reticulum (ER) is an important organelle for maintaining the homeostasis of eukaryotic cells. When the cells are damaged by excessive oxidation and other unfavorable factors, the ER function is affected, triggering ERS, and excessive or long-lasting ERS can cause an increase in ROS levels and induce apoptosis (45). Previous studies have confirmed that 5-FU can activate the ERS and induce the rise of ROS in colon cancer cells, and subsequently induces apoptosis through the mitochondrial pathway (20). In this study, CAP was observed to activate the ERS, reduce the mitochondrial potential, and increase ROS production *in vivo* and *in vitro*. Similarly, the differential expression of TP in T cells affected the CAP-induced changes *in vitro*. Subsequently, inhibition of ERS in T cells attenuated CAP-induced ROS generation and mitochondria-mediated apoptosis *in vitro*. These results indicate that ERS induction, ROS production, and mitochondria-mediated apoptosis activation may be the mechanisms behind CAP-induced T cell apoptosis. Certainly, T cell apoptosis and immunosuppressive effects are biologically relevant but, at the same time, different. Nonetheless, considering that the induction of T cell apoptosis is one of the mechanisms by which the classical immunosuppressive agents, such as tacrolimus and mycophenolate mofetil, exert their immunosuppressive effects (46–48), the exploration of T cell apoptosis induced by CAP may lay the foundation for further research into its immunosuppressive effects. Alternatively, for more practical application, the results of this experiment may provide the basis for the application of CAP in the treatment of TP-expressing lymphoid cancers.

**Figure 8:**
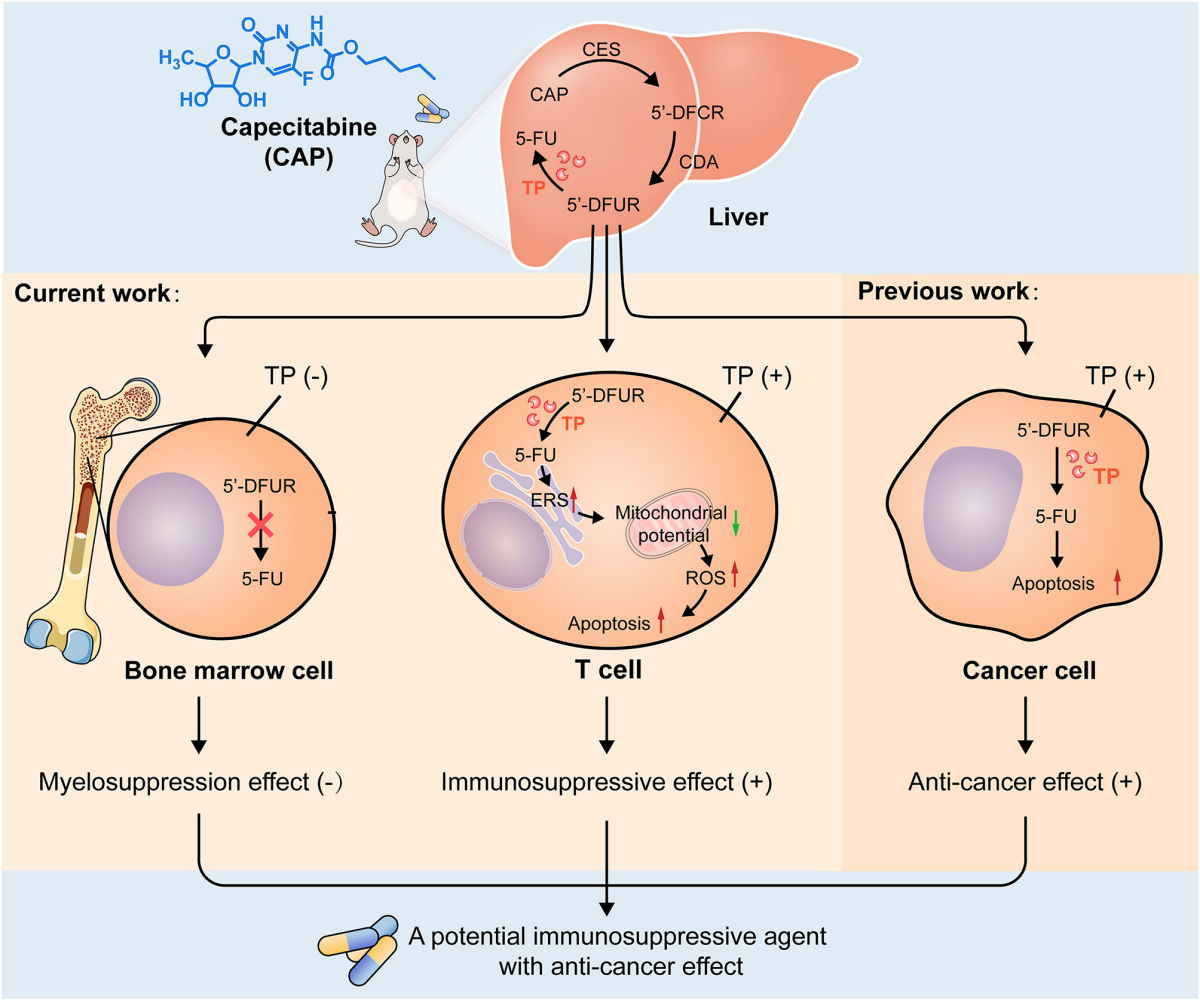
Possible mechanisms of CAP as a drug with both immunosuppressive and anti-cancer effects. After oral administration, CAP is converted into 5′DFCR and 5′DFUR in the liver by CES and CDA. There is no expression of TP in bone marrow tissue; thus, capecitabine cannot be converted into 5-FU, which circumvents myelosuppression and predicts the feasibility of its long-term medication. TP is expressed in T cells, and capecitabine can be converted into 5-FU there; thus, CAP increases endoplasmic reticulum stress response, reduces the membrane potential of mitochondria, increases the expression of ROS, finally, mitochondria-mediated apoptosis was induced. TP is also expressed in cancer cells, and CAP can induce cancer cell apoptosis and exert anti-cancer effect; this indicates that apoptosis is the final destination of CAP acting on T cells and cancer cells, both of which express TP, which lays an experimental foundation for exploring CAP as a drug with both immunosuppressive and anti-cancer effects.

In summary, the differential expression of TP in tissue and cells not only allows CAP to avoid the side effects of myelosuppression but it also allows CAP to induce T cell apoptosis. Apoptosis may be the final fate of CAP-treated T cells and cancer cells, both of which express TP; this lays the experimental foundation for exploring CAP as a new immunosuppressant with anti-cancer effect (**Figure 8**). Clearly, there is currently no clear evidence that CAP can prevent the recurrence of HCC. Therefore, it is worthy of further exploration whether CAP can effectively prevent the recurrence of HCC at the dose that can achieve immunosuppressive effect. Therefore, in the future, we will not only use acute rejection animal models to further confirm the immunosuppressive effect of CAP, but we will also attempt to establish a tumor-bearing animal model simultaneously bearing an organ allograft to confirm that CAP has both immunosuppressive and anti-cancer effects. In addition, we also plan to focus on the target T cell subsets of CAP, including Th1, Th2, Th9, Th17, Th22, Tfh, regulatory T cells, and CD8^+^cytotoxic T cells. In brief, the immunosuppressive effect of CAP deserves to be further explored in order to lay an experimental foundation for CAP as an immunosuppressant with anti-cancer effect to optimize the medication regimen for liver transplant patients with HCC. Meanwhile, the exploration of new immunosuppressant based on CAP, which is a pyrimidine nucleoside antimetabolite, will also lead the research direction of pyrimidine immunosuppressant in the field of organ transplantation.

## Data Availability Statement

The original contributions presented in the study are included in the article/**Supplementary Material**. Further inquiries can be directed to the corresponding authors.

## Ethics Statement

The animal study was reviewed and approved by Ethics Committee of Nankai University.

## Author Contributions

SaZ, ZW, HZ, and ZYS conceived the study. SaZ, ZW, HZ and ZYS designed the research. SaZ, SF, HW, LC, JW, SiZ, JL, and SL assisted in mouse experiments. SaZ, SF, TL, WH, LC, and JW performed the research. SaZ, ZYS, HZ, SYo, SYa, LL, WH, ZLS, and SL analyzed the data. ZW, HZ, and ZYS supervised the study. SaZ, ZW, SF, and TL wrote the paper. SYo, SYa, LL, HZ, and ZYS contributed to revision. All authors contributed to the article and approved the submitted version.

## Funding

This study was supported by grants from the National Key Research and Development Program of China (2020YFA0710802), the Youth Science Fund of the Nature Science Foundation of Tianjin (20JCQNJC01370), and the Science Foundation of Tianjin Health Commission (ZC20065, ZC20089).

## Conflict of Interest

The authors declare that the research was conducted in the absence of any commercial or financial relationships that could be construed as a potential conflict of interest.

## Publisher’s Note

All claims expressed in this article are solely those of the authors and do not necessarily represent those of their affiliated organizations, or those of the publisher, the editors and the reviewers. Any product that may be evaluated in this article, or claim that may be made by its manufacturer, is not guaranteed or endorsed by the publisher.
