# Supplementary figures and images for: Capecitabine Can Induce T Cell Apoptosis: A Potential Immunosuppressive Agent With Anti-Cancer Effect
Source: Front Immunol. 2021 Sep 7;12:737849. doi: 10.3389/fimmu.2021.737849 (PMC8452994; doi:10.3389/fimmu.2021.737849)

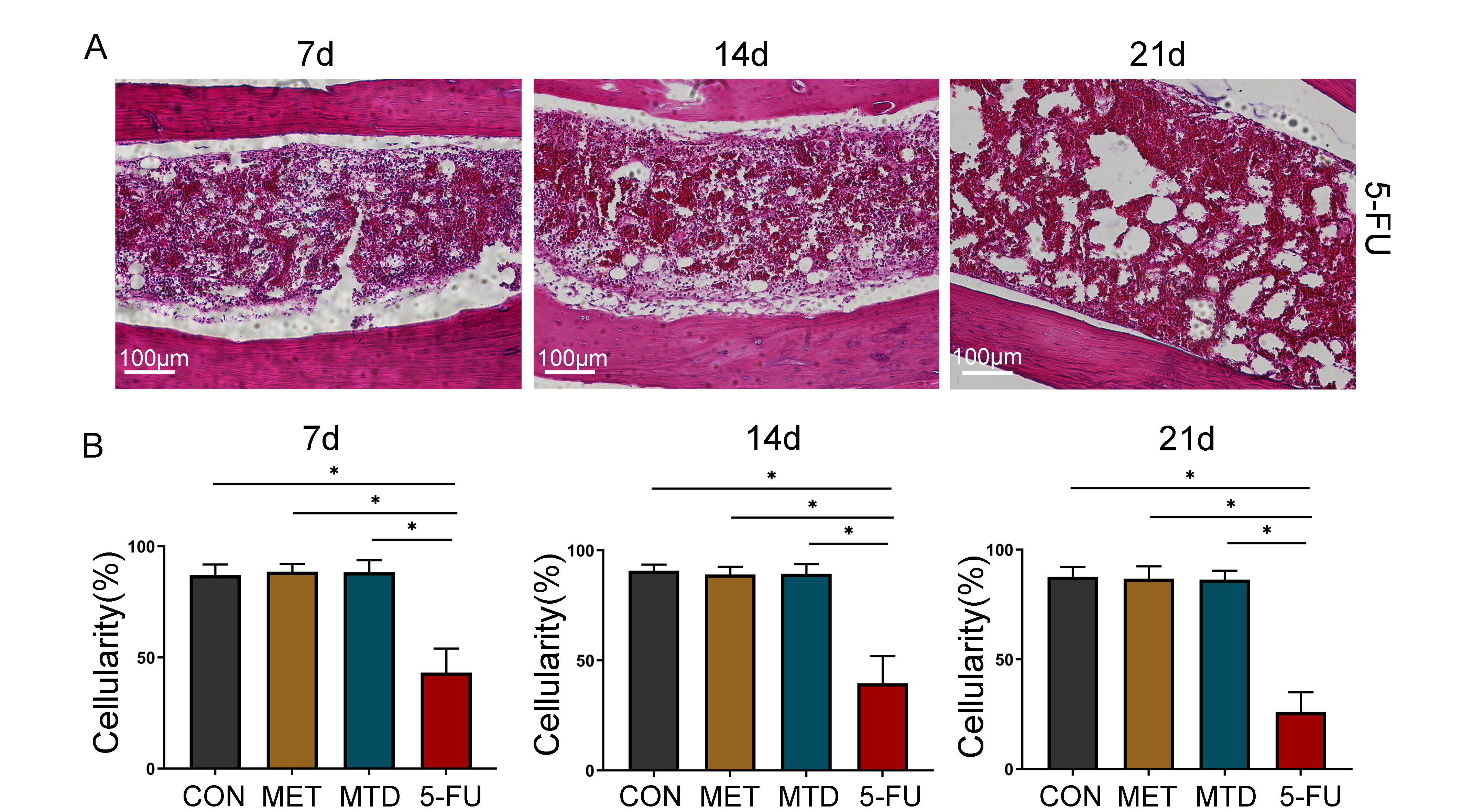

Supplement: Supplementary file 1 [file Image_1.jpeg]

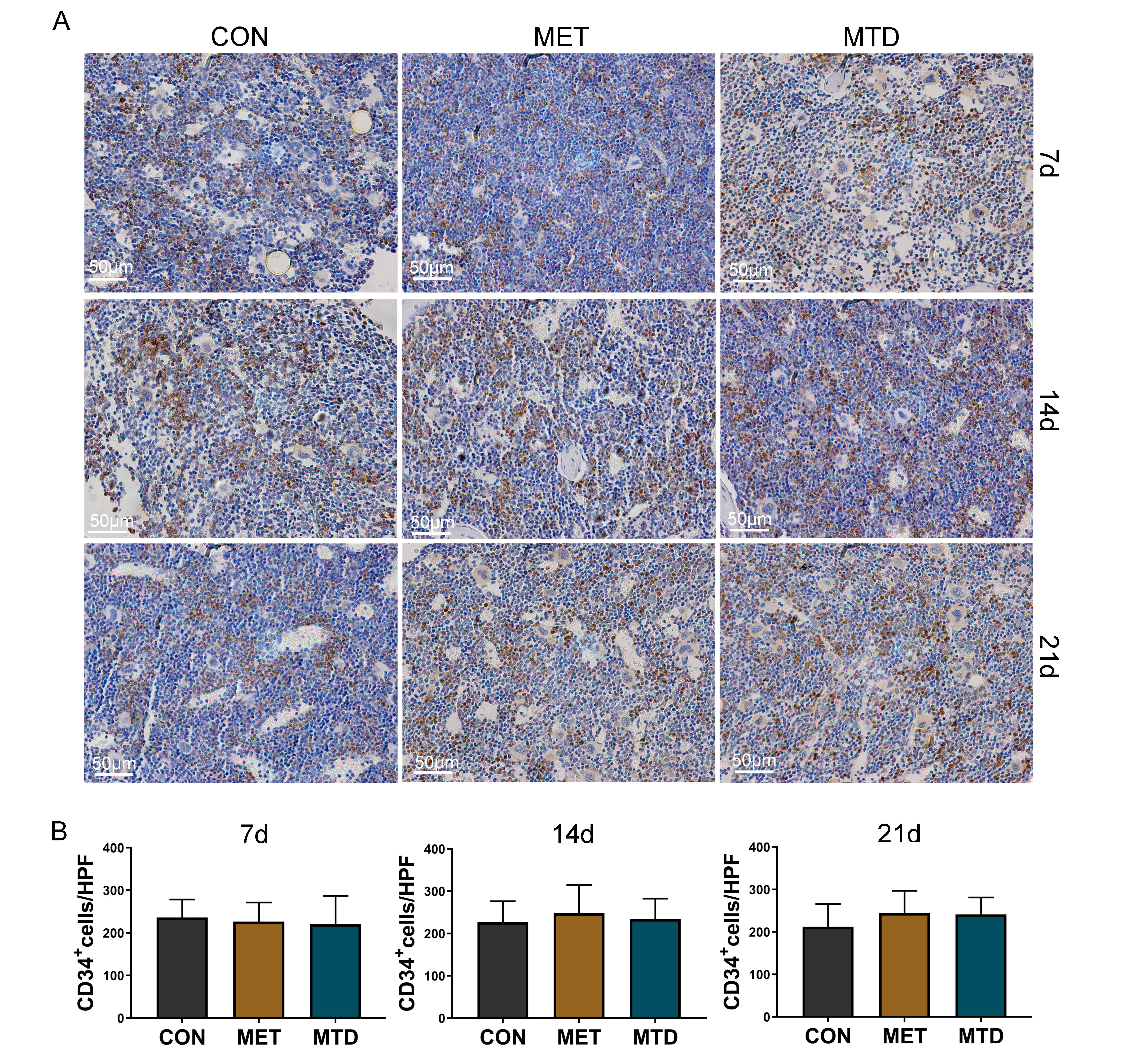

Supplement: Supplementary file 2 [file Image_2.jpeg]

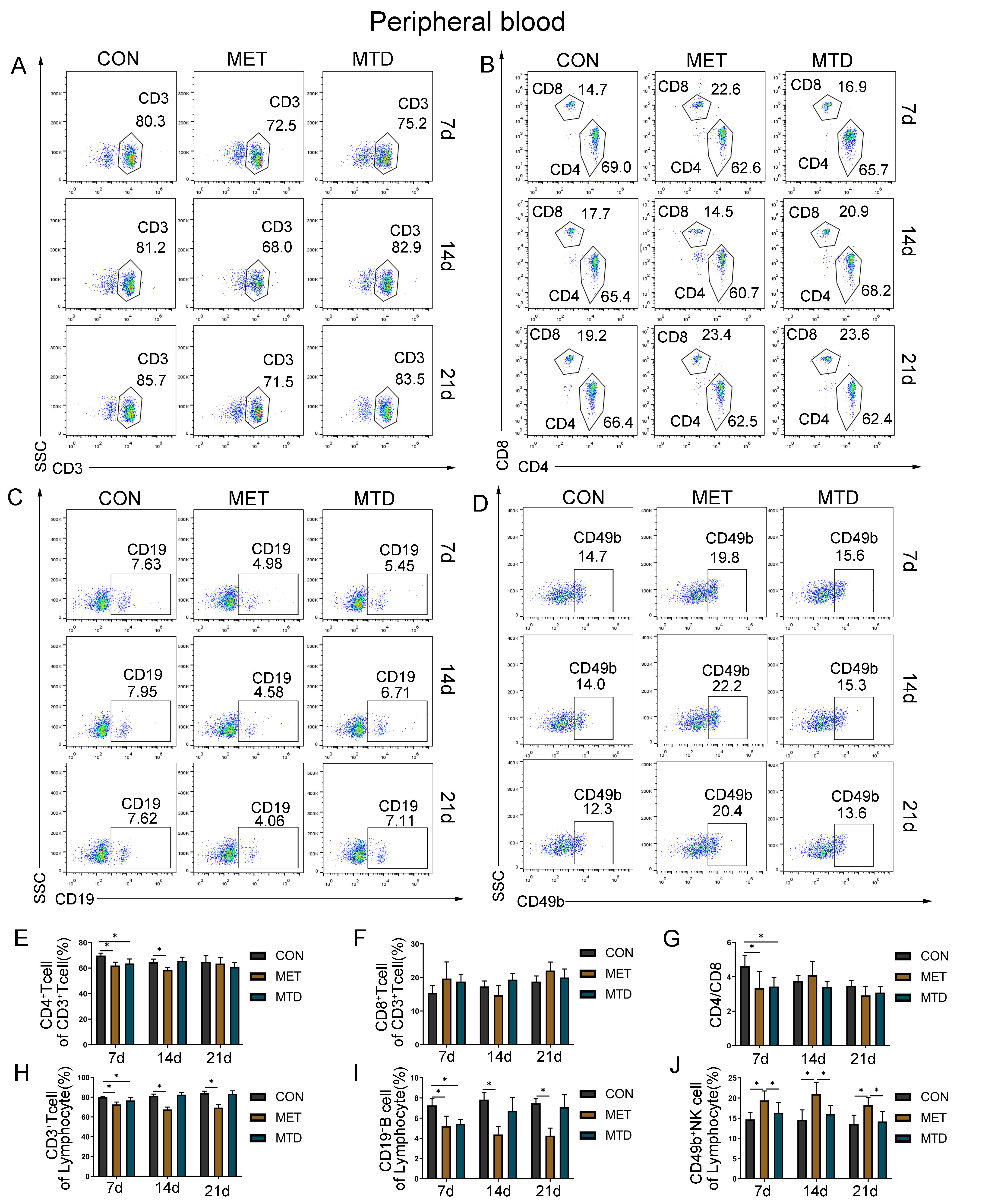

Supplement: Supplementary file 3 [file Image_3.jpeg]

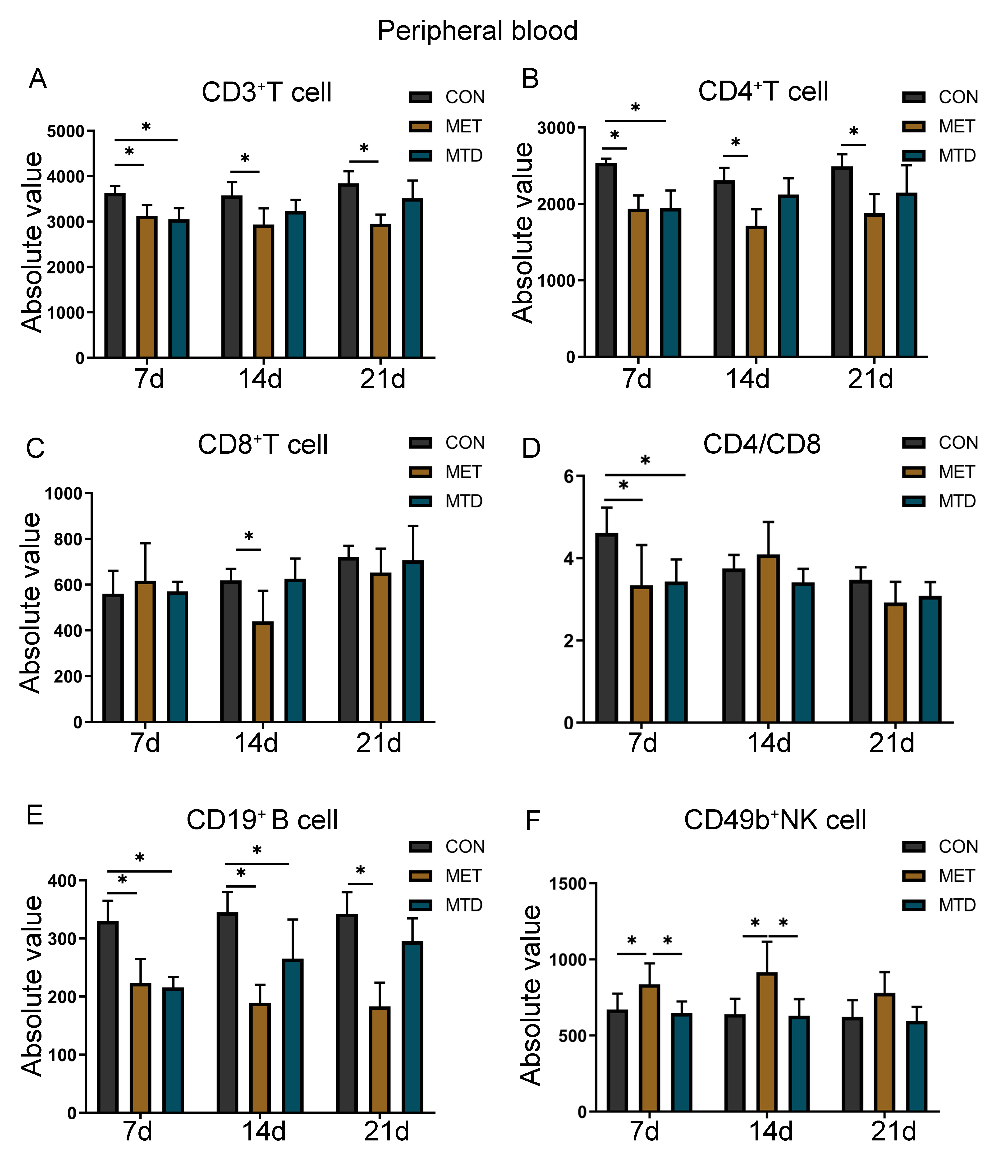

Supplement: Supplementary file 4 [file Image_4.jpeg]

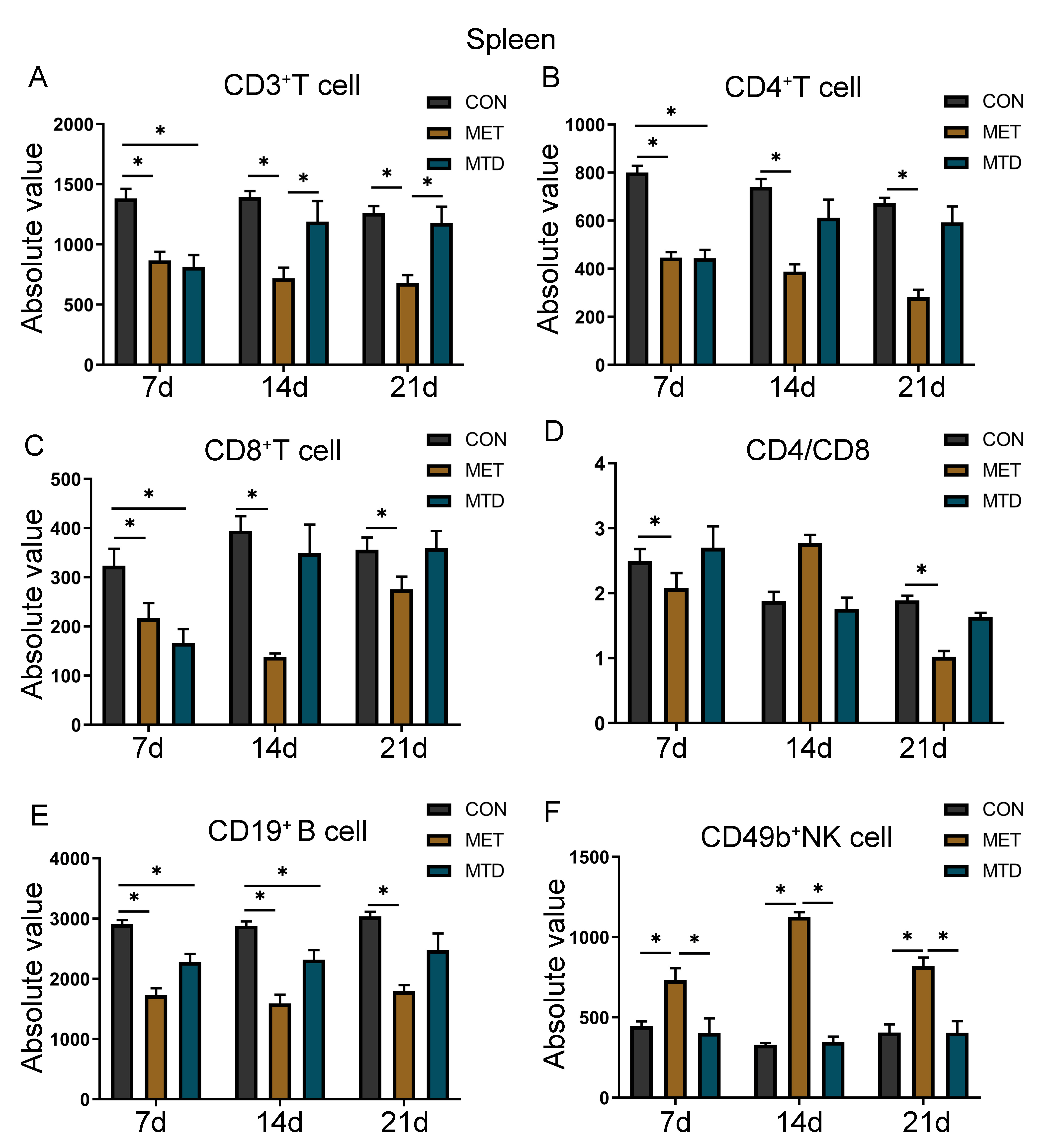

Supplement: Supplementary file 5 [file Image_5.jpeg]

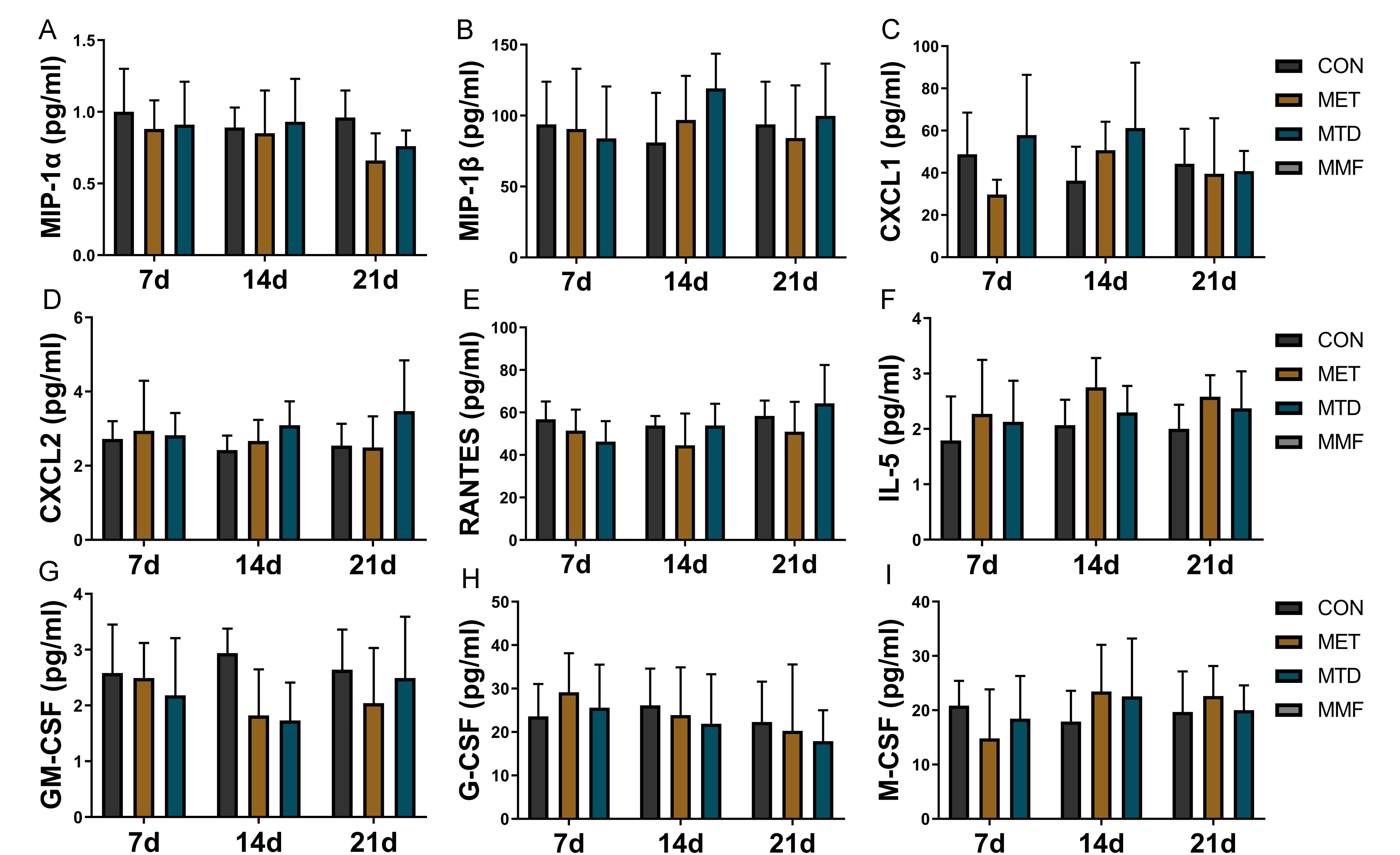

Supplement: Supplementary file 6 [file Image_6.jpeg]

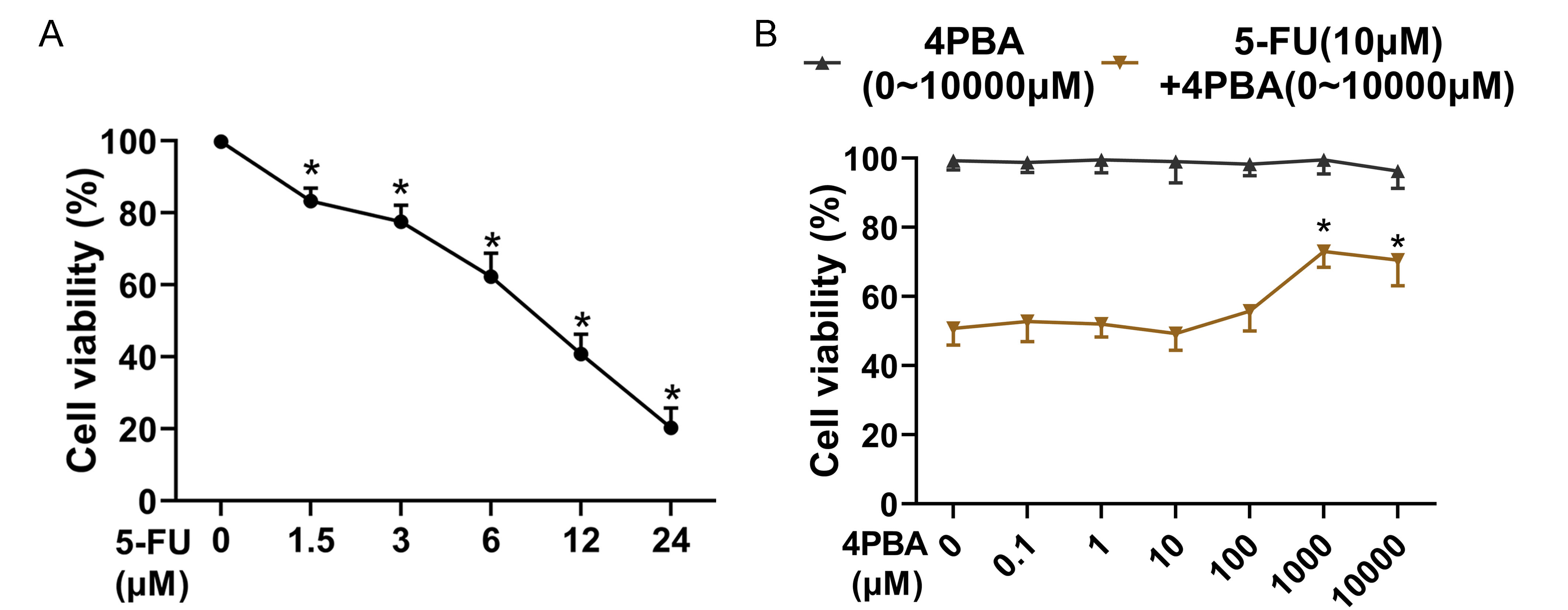

Supplement: Supplementary file 7 [file Image_7.jpeg]
